# Longitudinal outcomes of non-transplanted ornithine transcarbamylase deficiency in males and females: a multicentre UK natural history cohort

**DOI:** 10.1186/s13023-026-04517-w

**Published:** 2026-07-28

**Authors:** Berna Seker-Yilmaz, Julien Baruteau, Raphael Buttigieg, Anupam Chakrapani, Michael Champion, Catherine Davies, James Davison, Marjorie Dixon, Sherry Fang, Emma Footitt, Joanna Gribben, Stephanie Grunewald, Gemma Hack, Thomas Hartley, Hugh Lemonde, Philippa Mills, Andrew A. M. Morris, Helen Mundy, Dinusha Pandithan, Radha Ramachandran, Siyamini Sivananthan, Chern Tan, Dexter Tarr, Louise Van Dorp, Roshni Vara, Abigail Woodward, Paul Gissen

**Affiliations:** 1 Genetics and Genomic Medicine Department, Great Ormond Street Institute of Child Health, University College London, London, WC1N 1EH UK; 2 Department of Paediatric Inherited Metabolic Disease, Evelina Children’s Hospital, Guy’s, and St Thomas’ NHS Foundation Trust, London, SE1 7EH UK; 3 Department of Paediatric Metabolic Medicine, Great Ormond Street Hospital for Children NHS Foundation Trust, London, WC1N 3JH UK; 4 Department of Adult Inherited Metabolic Disease, Guy’s and St Thomas’ NHS Foundation Trust, London, SE1 7EH UK; 5 Dietetics, Great Ormond Street Hospital for Children NHS Foundation Trust, London, WC1N 3JH UK; 6 Willink Unit, Manchester Academic Health Sciences Centre, Genomic Medicine, Manchester University Hospitals NHS Foundation Trust, Oxford Road, Manchester, M13 9WL UK

**Keywords:** Ornithine transcarbamylase deficiency, Urea cycle disorder, Hyperammonaemia, Natural history cohort, Genotype–phenotype correlation, Neurodevelopment, Chronic management, Long-term outcomes

## Abstract

**Background:**

Ornithine transcarbamylase deficiency (OTCD) demonstrates marked phenotypic heterogeneity, ranging from severe neonatal hyperammonaemia to asymptomatic disease. While acute hyperammonaemic crises are well recognised, the long-term burden in medically managed, non-transplanted individuals across the phenotypic spectrum remains incompletely defined. This study aimed to characterise longitudinal clinical outcomes and disease burden in a multicentre cohort and to explore genotype–phenotype relationships.

**Results:**

Forty-five individuals were included (27 decompensated: 26 late-onset and 1 neonatal-onset; 18 non-decompensated), with median age at last follow-up of 13.3 years (IQR, 7.8–17.7). Molecular confirmation was available in 40/45 (88.9%). Among the decompensated cohort, 111 hyperammonaemic decompensation episodes, 224 hospital admissions, and 70 episodes requiring intravenous nitrogen-scavenger rescue were recorded; 16/26 (61.5%) late-onset individuals experienced recurrent decompensations. Individuals without documented decompensation also demonstrated clinically relevant morbidity, including behavioural problems (38.9%), recurrent vomiting (44.4%), chronic fatigue (33.3%), and learning difficulties (16.7%).

**Conclusion:**

In this multicentre cohort of non-transplanted individuals, OTCD was associated with substantial morbidity across the phenotypic spectrum. Late-onset disease was characterised by recurrent metabolic instability, frequent hospitalisation, and neurodevelopmental burden, while individuals without overt hyperammonaemic episodes also experienced clinically relevant symptoms. These findings highlight that clinically relevant disease burden extends across the OTCD phenotypic spectrum. Improved risk stratification and disease-modifying therapies remain important unmet needs.

## Background

Ornithine transcarbamylase (OTC) deficiency (OTCD) [MIM: 311250] is the most common urea cycle disorder (UCD) and is caused by pathogenic variants in the *OTC* gene, leading to impaired detoxification of nitrogen and accumulation of ammonia [1]. The prevalence of OTCD in the overall population has been estimated at approximately 1 in 62,000–70,000 live births [2–6]. 

OTCD is characterized by marked phenotypic heterogeneity. Hemizygous males may present with a neonatal-onset phenotype (≤ 28 days of age), typically associated with a complete absence of enzyme activity, and frequently caused by null variants, defined as variants predicted to result in no residual enzyme activity. Alternatively, individuals may present with a late-onset phenotype (> 28 days of age), which reflects partial residual enzyme activity and can occur in both males and heterozygous females [7, 8]. In heterozygous females, the clinical presentation is particularly variable owing to the random pattern of X-chromosome inactivation in hepatocytes; although many remain asymptomatic, approximately 20% develop symptoms, with highly diverse ages of onset and manifestations [9]. Across all phenotypes, acute hyperammonaemic decompensations may be precipitated by catabolic stressors and can occur at any age and may result in severe or life-threatening hyperammonaemic crises [10]. Beyond acute hyperammonaemic episodes, patients may experience chronic manifestations, including protein aversion, recurrent vomiting, developmental delay, neuropsychiatric disturbances, and varying degrees of liver dysfunction [9–11]. 

The diagnosis of OTCD is typically supported by characteristic biochemical abnormalities, while identification of a pathogenic *OTC* variant remains the diagnostic gold standard [12]. Up to 20% of clinically and biochemically suggestive cases do not have detectable coding region mutations, which necessitates additional investigations such as regulatory region sequencing or RNA-based analyses [13–15]. Standard management includes protein restriction, nitrogen scavengers and arginine or citrulline supplementation; however, these measures do not reliably prevent hyperammonaemic episodes, and therefore substantial therapeutic need persists [12]. Although liver transplantation remains the only definitive treatment, several investigational therapeutic approaches, including adeno-associated viral vector-mediated gene therapy, messenger RNA therapies, and genome editing strategies, are currently under development with the aim of restoring hepatic OTC function [16, 17]. 

Despite advances in diagnosis and treatment, the long-term neurological and metabolic trajectory of individuals with OTCD remains insufficiently defined, underscoring the need for contemporary and systematically collected natural history data [8, 18]. Existing long-term studies are constrained by small sample sizes, heterogeneous inclusion of UCDs and reliance on retrospective data, limiting their value for accurate counselling, risk stratification and the development of clinical trials for emerging therapies.

To address these limitations, we conducted a multicentre natural history study across three UK metabolic centres, integrating retrospective data from 1 January 2018 to 31 December 2020 with prospective follow-up to November 2025. This report focuses on non-transplanted patients and describes their clinical characteristics, neurocognitive outcomes, and therapeutic management. The primary aims were to define the spectrum of disease encountered in routine practice, quantify the burden and timing of hyperammonaemic decompensations and major neurological outcomes, and examine demographic, genotypic, and clinical determinants of disease course. The complementary cohort of liver-transplanted individuals has been reported separately, enabling the present analysis to characterise longitudinal outcomes in patients managed without transplantation [16]. 

## Methods

### Study design and setting

We conducted a multicentre observational cohort study of individuals with OTCD managed at three UK specialist inherited metabolic disease centres: Guy’s and St Thomas’ NHS Foundation Trust, Great Ormond Street Hospital for Children NHS Foundation Trust, and Manchester University NHS Foundation Trust. The study comprised a retrospective phase from 1 January 2018 to 31 December 2020 and a prospective phase embedded within routine clinical follow-up from 1 January 2021 to 1 November 2025. Clinical data were collected using a harmonised natural history case report form implemented across all centres. No study-specific investigations or interventions were introduced.

### Participants

Individuals were eligible if they were under follow-up at one of the participating centres and had a genetically and/or biochemically confirmed diagnosis of OTCD. Genetic confirmation required identification of a pathogenic or likely pathogenic variant in the OTC gene. In the absence of molecular confirmation, biochemical diagnosis required documented hyperammonaemia with a plasma amino acid profile consistent with proximal urea cycle dysfunction and elevated urinary orotic acid, with exclusion of alternative urea cycle disorders. Across the three participating centres, 65 individuals with OTCD were under active follow-up during the study period. Twenty patients underwent liver transplantation and were therefore excluded from the present analysis, leaving 45 non-transplanted individuals for inclusion. Female participants identified through family screening were included when considered clinically affected individuals requiring specialist metabolic follow-up. Unaffected carrier relatives without clinical follow-up were not included.

### Phenotypic classification and analytic cohorts

Phenotype was defined according to age at first hyperammonaemic decompensation as neonatal-onset (≤ 28 days of life), late-onset (> 28 days of life), or non-decompensated. Individuals with no documented hyperammonaemic decompensations at any time were classified as non-decompensated. Presymptomatically identified individuals were classified according to the age at their first documented hyperammonaemic decompensation.

A hyperammonaemic decompensation was defined as a symptomatic episode with plasma ammonia ≥ 100 µmol/L (above the upper limit of normal at participating laboratories, typically < 50 µmol/L) requiring medical evaluation and/or treatment; multiple ammonia measurements obtained during a single clinical episode or hospital admission were considered part of the same event. Recurrent hyperammonaemic decompensations were defined as two or more distinct episodes during follow-up.

For longitudinal analyses, participants were additionally stratified into decompensated and non-decompensated cohorts. The decompensated cohort comprised all individuals with at least one documented hyperammonaemic decompensation at any point during their clinical course, including both neonatal-onset and late-onset phenotypes. The non-decompensated cohort comprised individuals with no documented hyperammonaemic decompensations throughout the observation period and no recorded history of decompensation before study entry. Individuals classified as non-decompensated should not be considered unaffected carriers. All remained under specialist metabolic follow-up because of molecular diagnosis, biochemical abnormalities, clinical symptoms, or recognised future risk of metabolic decompensation.

### Growth assessment

Anthropometric data were extracted from clinical records. Age- and sex-adjusted z-scores for weight, height, and BMI were calculated using WHO and UK reference standards. In paediatric patients, underweight and stunting were defined as BMI-for-age and height-for-age z-scores < − 2, respectively, while overweight and obesity were defined as BMI-for-age z-scores > + 1 and > + 2, respectively. In adults, overweight and obesity were defined as BMI ≥ 25 kg/m² and ≥ 30 kg/m², respectively. The neonatal-onset individual was excluded from inferential growth analyses and described separately.

### Neurocognitive and imaging outcomes

Neurocognitive outcomes were recorded from formal age-appropriate psychometric assessments where available. In the absence of standardised testing, functional status was determined from multidisciplinary clinical documentation. Educational placement in paediatric patients was classified according to level of support required. Documented behavioural or neurodevelopmental diagnoses were captured. Brain MRI findings were recorded where performed and categorised according to radiology reports.

### Therapeutic management

Therapeutic data included prescribed dietary protein intake, essential amino acid (EAA) supplementation, use of specialised low-protein food products, nitrogen-scavenger therapy (sodium benzoate, sodium phenylbutyrate, glycerol phenylbutyrate), arginine and citrulline supplementation, enteral feeding support, provision of emergency regimen for illness management, and vitamin and mineral supplementation.

### Statistical analysis

Analyses were performed at the patient level. Continuous variables are presented as mean (standard deviation) or median (interquartile range), as appropriate, and categorical variables as frequencies and percentages. Comparisons between phenotypic groups were conducted using χ² or Fisher’s exact tests for categorical variables and Mann–Whitney U or Kruskal–Wallis tests for continuous variables, as appropriate. The neonatal-onset individual was excluded from inferential comparisons. Analyses were based on available data, and no imputation for missing values was performed.

## Results

### Cohort characteristics

Forty-five individuals (27 females, 18 males) with OTCD were included in this multicentre observational cohort. All individuals were alive and non-transplanted at last follow-up, and.

no deaths occurred during the study follow-up period. Patients were managed at three UK specialist inherited metabolic disease centres: Guy’s and St Thomas’ NHS Foundation Trust (*n* = 38), Great Ormond Street Hospital for Children NHS Foundation Trust (*n* = 5), and Manchester University NHS Foundation Trust (*n* = 2).

At the predefined prospective study end date of 1 November 2025, age at last follow-up ranged from 2 months to 25.6 years, with a median age of 13.3 years (interquartile range [IQR] 7.8–17.7 years).

### Follow-up structure and duration

Follow-up was predominantly longitudinal, with most participants contributing data across both retrospective and prospective study phases (Table 1). Thirty-four individuals (75.6%) had data available from both the retrospective period (1 January 2018 to 31 December 2020) and the prospective follow-up phase (1 January 2021 to 1 November 2025). A smaller proportion contributed data exclusively during the retrospective phase (*n* = 3, 6.7%) or exclusively during the prospective phase (*n* = 8, 17.8%).

Median duration of retrospective follow-up was 3.0 years (IQR, 2.4–3.0 years), and median duration of prospective follow-up was 4.8 years (IQR, 2.2–4.8 years).

### Transition to adult services

During the study period, 16 individuals (35.6%) transitioned from paediatric to adult metabolic services. Transition occurred within the context of ongoing longitudinal follow-up and reflects routine clinical practice at participating centres.

**Table 1:** Baseline characteristics, follow-up, and transition status of the OTCD cohort

Characteristic	Value
**Total number of individuals**	45
**Female**,** n (%)**	27 (60.0)
**Male**,** n (%)**	18 (40.0)
**Specialist Centre**,** n (%)**	
Guy’s and St Thomas’ NHS Foundation Trust	38 (84.4)
Great Ormond Street Hospital for Children NHS Foundation Trust	5 (11.1)
Manchester University NHS Foundation Trust	2 (4.4)
**Age at last follow-up**,** years**	
Median (IQR)	13.3 (7.8–17.7)
Range	0.16–25.6
**Follow-up structure**,** n (%)**	
Retrospective and prospective	34 (75.6)
Retrospective only	3 (6.7)
Prospective only	8 (17.8)
**Follow-up duration**,** years**	
Retrospective, median (IQR, years)	3.0 (2.4–3.0)
Prospective, median (IQR, years)	4.8 (2.2–4.8)
Total follow-up, range (years)	0.16–7.8
**Transitioned to adult services**,** n (%)**	16 (35.6)

### Phenotype and diagnostic pathways

The cohort comprised 26 individuals (57.8%) with late-onset OTCD, 18 individuals (40.0%) who were non-decompensated, and one individual (2.2%) with neonatal-onset disease.

Sex distribution differed across phenotypes. The neonatal-onset individual was male. Among individuals with late-onset OTCD, 17 were female and 9 were male, whereas the non-decompensated cohort comprised 10 females and 8 males. As expected for an X-linked disorder, females were more frequently represented among non-decompensated individuals identified through family screening, although clinically significant disease was observed in both sexes.

Diagnostic evaluation was initiated following acute hyperammonaemic presentation in 22 of 45 individuals (48.9%), family history–based cascade testing in 22 individuals (48.9%), and newborn screening in one individual (2.2%), identified through the UK Newborn Genomes Programme (Generation Study).

Among individuals with late-onset OTCD, the majority (21 of 26, 80.8%) were diagnosed following an acute hyperammonaemic presentation, while five individuals (19.2%) were identified through family history-based pathways, including perinatal diagnosis in several cases. In contrast, non-decompensated individuals were almost exclusively diagnosed through family history-based cascade testing (17 of 18, 94.4%), with a single non-decompensated individual identified through newborn genomic screening.

Age at diagnosis was available for all individuals. Among those diagnosed through family history–based pathways (*n* = 22), eight individuals (36.4%) were diagnosed perinatally because of known familial risk, while the remaining 14 individuals (63.6%) were diagnosed postnatally through cascade testing later in infancy or childhood. For those diagnosed postnatally via family history, the median age at diagnosis was 76 months (interquartile range [IQR] 47.5–121 months, range 8–160 months). Individuals diagnosed following acute clinical presentation had a median age at diagnosis of 26 months (IQR, 13–48 months, range 0.22–70 months). The individual identified through newborn screening was diagnosed at 1.5 months of age while clinically non-decompensated.

Review of longitudinal clinical records demonstrated that 8 of 21 individuals with late-onset OTCD diagnosed following acute presentation (38.1%) had experienced chronic or subtle symptoms prior to their first documented hyperammonaemic decompensation. These included recurrent vomiting, feeding difficulties or protein aversion, developmental delay, autism spectrum disorder or behavioural symptoms, and other non-specific neurological features that were not initially recognised as indicative of an underlying UCD. All eight individuals experienced a diagnostic delay, with a median delay of 2.5 months (IQR, 2.0–7.5 months, range 1–21 months).

### Clinical presentation and hyperammonaemic burden

Among the 27 individuals in the decompensated cohort, 22 (81.5%) presented with acute metabolic decompensation at diagnosis. At first decompensation, vomiting was the most frequent presenting symptom, occurring in 23/27 individuals (85.2%), followed by lethargy or reduced consciousness in 22/27 (81.5%). Seizures were documented in 4/27 individuals (14.8%), and acute liver failure or severe liver dysfunction was present in 8/27 (29.6%). Less frequently reported features included headache, feeding intolerance, and encephalopathic behavioural change.

Peak plasma ammonia concentrations at first presentation were markedly elevated. Among late-onset individuals, the median peak ammonia concentration was 249 µmol/L (IQR, 188–317 µmol/L, range 98–3000 µmol/L). Severe hyperammonaemia (≥ 300 µmol/L) occurred in 7/26 late-onset individuals (26.9%), including two individuals (7.7%) with extreme elevations ≥ 700 µmol/L. The neonatal-onset individual presented with catastrophic hyperammonaemia (> 2000 µmol/L) in association with multisystem involvement.

Of the 27 individuals in the decompensated cohort, 5 (18.5%) were diagnosed presymptomatically through family screening and subsequently developed symptoms during longitudinal follow-up.

Acute liver failure at first presentation was observed in 8/27 individuals in the decompensated cohort (29.6%), including 7/26 late-onset patients and the neonatal case. Among late-onset individuals with hepatic involvement at presentation, 2/7 (28.6%) experienced recurrent hyperammonaemic episodes during follow-up.

**Table 2:** Longitudinal hyperammonaemic burden in the decompensated OTCD cohort

Clinical subgroup	*n*	Hyperammonaemic decompensations, *n* (mean per patient)	Peak ammonia during follow-up, median (range), µmol/L	Hospital admissions, *n* (mean per patient)	Hyperammonaemic decompensations requiring IV scavenger rescue, *n* (mean per patient)
**Decompensated OTCD cohort (overall)**	**27**	**111 (4.1)**	**262 (105–453)**	**224 (8.3)**	**70 (2.6)**
Neonatal-onset	1	29 (29.0)	374	43 (43.0)	10 (10.0)
Late-onset, clinically presenting	21	66 (3.1)	231 (105–453)	146 (7.0)	49 (2.3)
Late-onset, presymptomatically identified	5	16 (3.2)	262 (197–274)	35 (7.0)	11 (2.2)

Despite sustained dietary and pharmacological management, hyperammonaemic morbidity remained substantial in the decompensated OTCD cohort during longitudinal follow-up (Table 2). Across the decompensated cohort, 224 hospital admissions and 111 hyperammonaemic decompensations were recorded, corresponding to a mean of 8.3 admissions and 4.1 decompensations per patient. Intravenous nitrogen-scavenger rescue therapy was required in 70 episodes, demonstrating persistent metabolic instability despite maintenance treatment. Peak ammonia concentrations during follow-up ranged from 105 to 453 µmol/L, with a median of 262 µmol/L.

Morbidity was greatest in the neonatal-onset phenotype, in which one individual experienced 29 hyperammonaemic decompensation episodes, 43 hospital admissions, and 10 episodes requiring intravenous scavenger rescue. Among individuals with late-onset OTCD diagnosed following an initial hyperammonaemic decompensation, 66 hyperammonemic decompensations and 146 hospital admissions were recorded during follow-up, corresponding to mean rates of 3.1 decompensations and 7.0 admissions per patient. In this subgroup, the median peak plasma ammonia concentration during follow-up was 231 µmol/L (range, 105–453 µmol/L).

Individuals with late-onset OTCD identified presymptomatically through family screening also demonstrated substantial morbidity during follow-up. Across five individuals, 16 hyperammonaemic decompensations and 35 hospital admissions were recorded, corresponding to mean rates of 3.2 decompensations and 7.0 admissions per patient; 11 decompensations required intravenous nitrogen-scavenger rescue therapy. Peak plasma ammonia concentrations in this subgroup were similar to those observed in individuals diagnosed after an initial hyperammonaemic decompensation (median, 262 µmol/L; range, 197–274 µmol/L). Notably, all presymptomatically identified individuals subsequently developed hyperammonaemic decompensation, and four of five experienced recurrent decompensations, indicating that presymptomatically identified individuals still experienced substantial metabolic morbidity during follow-up.

No hyperammonaemic episodes were documented in non-decompensated individuals. However, 8/18 (44.4%) reported recurrent vomiting, predominantly during intercurrent infections, and 6/18 (33.3%) reported chronic fatigue, particularly in those aged > 18 years. All individuals were managed with emergency metabolic regimens, and none of these episodes were associated with biochemical hyperammonaemia.

Longitudinal plasma amino acid measurements, including glutamine+glutamate (Glx), arginine, and citrulline, were analysed using within-patient median values to account for repeated sampling (Table 3). Individuals with late-onset disease demonstrated higher plasma Glx concentrations compared with non-decompensated individuals (median 945.0 µmol/L [IQR, 916.0–1056.5] vs. 760.5 µmol/L [IQR 703.5–882.5]).

Median plasma arginine concentrations were higher in individuals with late-onset disease than in the non-decompensated cohort (67.8 µmol/L [IQR, 59.6–81.0] vs. 53.5 µmol/L [IQR, 44.0–71.2]); however, this difference was observed in the context of frequent therapeutic arginine supplementation in the decompensated cohort and therefore likely reflects treatment exposure rather than intrinsic metabolic differences.

Median plasma citrulline concentrations were comparable between groups (19.0 µmol/L [IQR, 15.0–29.5] vs. 17.5 µmol/L [IQR, 14.8–19.2]), although inter-individual variability was greater in the late-onset cohort, as reflected by a wider distribution of values.

The neonatal-onset individual exhibited marked longitudinal biochemical disturbance, characterised by persistently elevated Glx (median 1579.0 µmol/L) and very low citrulline (median 3.0 µmol/L), with arginine concentrations within the lower range observed in late-onset disease.

**Table 3:** Longitudinal plasma aminoacid profiles by phenotype

Phenotype	Total *n* in cohort	Glutamine+Glutamate (Glx), µmol/L median (IQR)	Arginine, µmol/L median (IQR)	Citrulline, µmol/L median (IQR)
Non-decompensated	18	760.5 (703.5–882.5)	53.5 (44.0–71.2)	17.5 (14.8–19.2)
Late-onset	26	945.0 (916.0–1056.5)	67.8 (59.6–81.0)	19.0 (15.0–29.5)
Neonatal	1	1579.0	38.0	3.0

Values are presented as median (interquartile range) unless otherwise indicated. Glx denotes the combined concentration of glutamine and glutamic acid. Age-specific reference ranges for plasma amino acids (µmol/L), derived from validated clinical laboratory data, are as follows

Glutamine: 263–776 (0–6 months); 279–695 (≥ 6 months across all older age groups)

Glutamic acid: 26–232 (0–6 months); 20–180 (≥ 6 months across all older age groups)

Arginine: 4–119 (0–1 month); 17–149 (≥ 1 month across all older age groups)

Citrulline: 5–36 (0–1 month); 5–41 (1–6 months); 8–46 (6–12 months); 10–51 (≥ 1 year across all older age groups)

### Chronic management and dietary treatment

All individuals in the cohort were managed within a specialist metabolic service, with regular clinical review and biochemical monitoring, and were provided with emergency treatment regimens for use during intercurrent illness. Long-term management strategies differed substantially by phenotype and were predominantly implemented in the decompensated cohort (late-onset and neonatal-onset disease; *n* = 27).

Dietary protein intake was actively managed in all individuals in the decompensated cohort (27/27, 100%) as part of long-term metabolic treatment. Quantitative assessment of dietary adequacy was feasible in only a subset. Dietary protein intake could be formally evaluated in 24 of 27 individuals in the decompensated cohort, while 3 individuals were categorised as “not formally counting” and excluded from assessment of safe protein intake for age according to WHO/FAO/UNU 2007 because of incomplete or unreliable dietary data.19 Among the 24 evaluable patients, 17/24 (70.8%) achieved age-appropriate safe protein intake targets, whereas 7/24 (29.2%) did not despite input from a specialist metabolic dietitian; no consistent association with age or documented feeding difficulties was identified.

Total prescribed protein intake among evaluable individuals in the decompensated cohort ranged from 0.45 to 1.29 g/kg/day, with a median of 0.95 g/kg/day (IQR, 0.69–1.11). Where documented, natural protein intake ranged from 0.34 to 1.29 g/kg/day, with a median of 0.62 g/kg/day (IQR, 0.59–1.00). In selected cases, intake was supplemented with essential amino acid (EAA) mixtures. EAA supplementation was used in 6 of 27 individuals in the decompensated cohort (22.2%), all of whom had natural protein intake below safe intake (SI), indicating use to compensate for inadequate dietary protein. Documented EAA intake ranged from 0.11 to 0.30 g/kg/day, with a median of 0.15 g/kg/day (IQR, 0.13–0.16). All individuals in the decompensated cohort receiving protein-restricted diets were additionally prescribed routine multivitamin and mineral supplementation.

Use of specialised low-protein foods was recorded in 10 of 24 individuals (41.6%) with available documentation, most commonly among children diagnosed in early childhood and those with recurrent hyperammonaemic decompensations.

A vegetarian dietary preference was documented in one non-decompensated female identified through family screening, adopted from late childhood, without subsequent hyperammonaemic decompensations or requirement for ammonia-scavenger therapy. Additionally, protein aversion was reported in four other non-decompensated individuals (two females and two males).

In individuals with persistent difficulty maintaining adequate oral intake or recurrent hyperammonaemic decompensations, escalation of nutritional support was required. Percutaneous endoscopic gastrostomy (PEG) feeding was used in 10 of 27 individuals in the decompensated cohort (37.0%), including 9 of 26 individuals with late-onset disease and the single neonatal-onset patient.

Central venous access via a totally implantable venous access device (Port-a-Cath) was present in 6 of 27 individuals in the decompensated cohort (22.2%), including 5 of 26 individuals with late-onset disease and the neonatal-onset patient. Concomitant use of PEG feeding and Port-a-Cath was observed in 5 of 27 individuals in the decompensated cohort (18.5%).

Chronic nitrogen-scavenger therapy was prescribed only for the decompensated cohort, and all 27 individuals received long-term treatment, including all 26 late-onset patients and the single neonatal-onset patient. Sodium benzoate was used in 21/27 patients (77.8%), phenylbutyrate in 18/27 (66.7%), and combination therapy in 21/27 (77.8%). Scavenger monotherapy was administered in 6/27 individuals (22.2%), consisting of 5 patients receiving sodium benzoate alone and 1 receiving phenylbutyrate alone.

Arginine supplementation was used in 23/27 individuals in the decompensated cohort (85.2%), while citrulline was prescribed in 16/27 (59.3%). In non-decompensated individuals (*n* = 18), amino acid supplementation was uncommon, with arginine used in 3/18 (16.7%), and no use of citrulline or combined arginine and citrulline therapy observed.

Among patients receiving scavenger monotherapy (*n* = 6), arginine was used in 4/6 (66.7%), citrulline in 1/6 (16.7%), and 1/6 (16.7%) received no amino acid supplementation.

Among patients receiving combination (dual) scavenger therapy (*n* = 21), arginine was used in 19/21 (90.5%) and citrulline in 15/21 (71.4%). Combined arginine and citrulline therapy were used in 15/21 (71.4%), while 4/21 (19.0%) received arginine alone. No patients received citrulline alone or no amino acid supplementation.

### Genotype-phenotype correlation

Genetic testing was available for all 45 individuals included in the cohort. A pathogenic or likely pathogenic *OTC* variant was identified in 40 of 45 individuals (88.9%), while no causative variant was detected in 5 late-onset patients (11.1%) despite a clinical and biochemical phenotype consistent with OTCD. In these five variant-negative cases, whole genome sequencing (WGS) did not identify a definitive disease-causing variant.

The molecular spectrum was heterogeneous and comprised missense variants, truncating variants, splice-altering variants, intronic variants, exon-level deletions, regulatory/promoter variants, as well as cases in which no pathogenic variant was identified (Table 4). Missense variants predominated, accounting for 32 of 45 individuals (71.1%), and were observed across all phenotypes. Variants predicted to confer greater functional disruption, including nonsense variants, a canonical splice-site variant, a deep intronic splice-altering variant, an in-frame deletion, and an exon-level deletion, were observed exclusively in the decompensated cohort (late-onset disease and the single neonatal-onset case) and were not identified in non-decompensated individuals (Table 4).

Late-onset disease exhibited the widest molecular diversity, encompassing all large deletions, truncating, splice-altering, and intronic variants identified in the cohort. Non-decompensated individuals (*n* = 18) demonstrated a narrower molecular spectrum (Table 4). Variants in this group were largely restricted to missense single-nucleotide variants (16/18, 88.8%) and regulatory/promoter variants (2/18, 11.1%). Recurrent missense variants, most notably c.240G > T (p.Lys80Asn) and c.118 C > T (p.Arg40Cys), were observed in multiple non-decompensated individuals from related families, consistent with ascertainment through family-based cascade testing.

Inspection of recurrent variants revealed a consistent sex-dependent pattern of clinical expression. Several missense variants were identified in non-decompensated females while being associated with late-onset disease in male carriers. Examples include c.240G > T (p.Lys80Asn), and c.867G > A, which were observed in non-decompensated females but in males presenting with late-onset OTCD. The single individual with neonatal-onset OTCD carried a missense variant (c.674 C > T; p.Pro225Leu) that was also identified in a female individual with late-onset disease. This pattern was consistent with X-linked inheritance and variable expression in heterozygous females.

A regulatory/promoter variant (c.-106 C > A) was identified in two male siblings from the same family. The older sibling presented neonatally, at approximately 2 days of age, with severe hyperammonaemia. At the time, targeted urea cycle gene panel testing did not identify a pathogenic variant, and the clinical course was initially interpreted as neonatal transient hyperammonaemia. He remained clinically stable on an unrestricted diet without ammonia scavenger therapy for six years, with use of an established emergency regimen during intercurrent illness. Following the subsequent birth of a younger male sibling, whole-genome sequencing performed through the UK Newborn Genomes Programme (Generation Study) identified the c.-106 C > A promoter variant, which was subsequently confirmed in the older sibling. Both individuals have remained non-decompensated during follow-up.

Two patients underwent measurement of hepatic OTC enzymatic activity. In one late-onset male patient, in whom no pathogenic variant was identified by genetic testing, enzymatic analysis demonstrated activity below the normal reference range. The second patient was a late-onset female carrying the c.662–663 + 1delinsGA variant; her measured OTC enzymatic activity was 6.3 mmol/h/mg protein (reference range 13–83 mmol/h/mg protein).

No novel pathogenic variants were identified within the cohort; all detected variants have been previously reported in OTCD or are consistent with established mutational mechanisms.

**Table 4:** Distribution of *OTC* gene variants by molecular class and clinical phenotype

Variant	Variant class	No. of patients (*n*)	Phenotype of patients detected (sex, number)
c.583G > A	Missense	1	Late onset (female, 1)
c.240G > T	Missense	10	Non-decompensated (female, 8; male, 2)
c.274 C > T	Missense	1	Late onset (female, 1)
c.410 C > A	Missense	1	Late onset (female, 1)
c.830G > A	Missense	2	Late onset (male, 1); Non-decompensated (male, 1)
c.674 C > T	Missense	2	Late onset (female, 1); Neonatal (male, 1)
c.829 C > T	Missense	2	Late onset (male, 2)
c.444G > C	Missense	1	Late onset (female, 1)
c.122 A > G	Missense	1	Late onset (female, 1)
c.133 C > G	Missense	1	Late onset (female, 1)
c.533 C > T	Missense	1	Late onset (female, 1)
c.118 C > T	Missense	4	Non-decompensated (female, 1; male, 3)
c.867G > A	Missense	2	Late onset (male, 1); Non-decompensated (female, 1)
c.222G > T	Missense	1	Late onset (male, 1)
c.913 C > T	Missense	1	Late onset (female, 1)
c.1015G > A	Missense	1	Late onset (female, 1)
c.421 C > T	Nonsense (loss-of-function)	1	Late onset (female, 1)
c.173G > A	Nonsense (loss-of-function)	1	Late onset (female, 1)
c.662–663 + 1delinsGA	Canonical splice-site	1	Late onset (female, 1)
c.540 + 265G > A	Deep intronic (splice-altering)	1	Late onset (female, 1)
c.929_931del	In-frame deletion	1	Late onset (male, 1)
Exon 5 deletion	Exon-level deletion (CNV)	1	Late onset (female, 1)
c.-106 C > A	Regulatory / promoter	2	Non-decompensated (male, 2)
No variant identified	—	5	Late onset (male, 3; female, 2)

### Growth

Anthropometric data at most recent follow-up was available for 45 individuals with OTCD. After excluding a single neonatal case, analyses included 26 paediatric patients (< 18 years) and 18 adults (≥ 18 years), of whom 16 had BMI recorded (non-decompensated = 9; late-onset = 7).

In paediatric patients, weight-for-age z-scores at last follow-up ranged from − 3.69 to + 3.70 (median 0.26; mean 0.57 ± 1.59), and height-for-age z-scores ranged from − 1.65 to + 2.20 (median 0.13; mean 0.23 ± 0.97). Underweight (weight-for-age z < − 2) was observed in 3/26 children, all with late-onset disease, of whom 2 were also stunted. Stunting (height-for-age z < − 2) occurred in 3/26 children, exclusively in late-onset patients. Overweight (weight-for-age z > + 1) was present in 9/26 children (non-decompensated = 4; late-onset = 5), with obesity (z > + 2) in 5/26 (non-decompensated = 2; late-onset = 3).

**Fig. 1:**
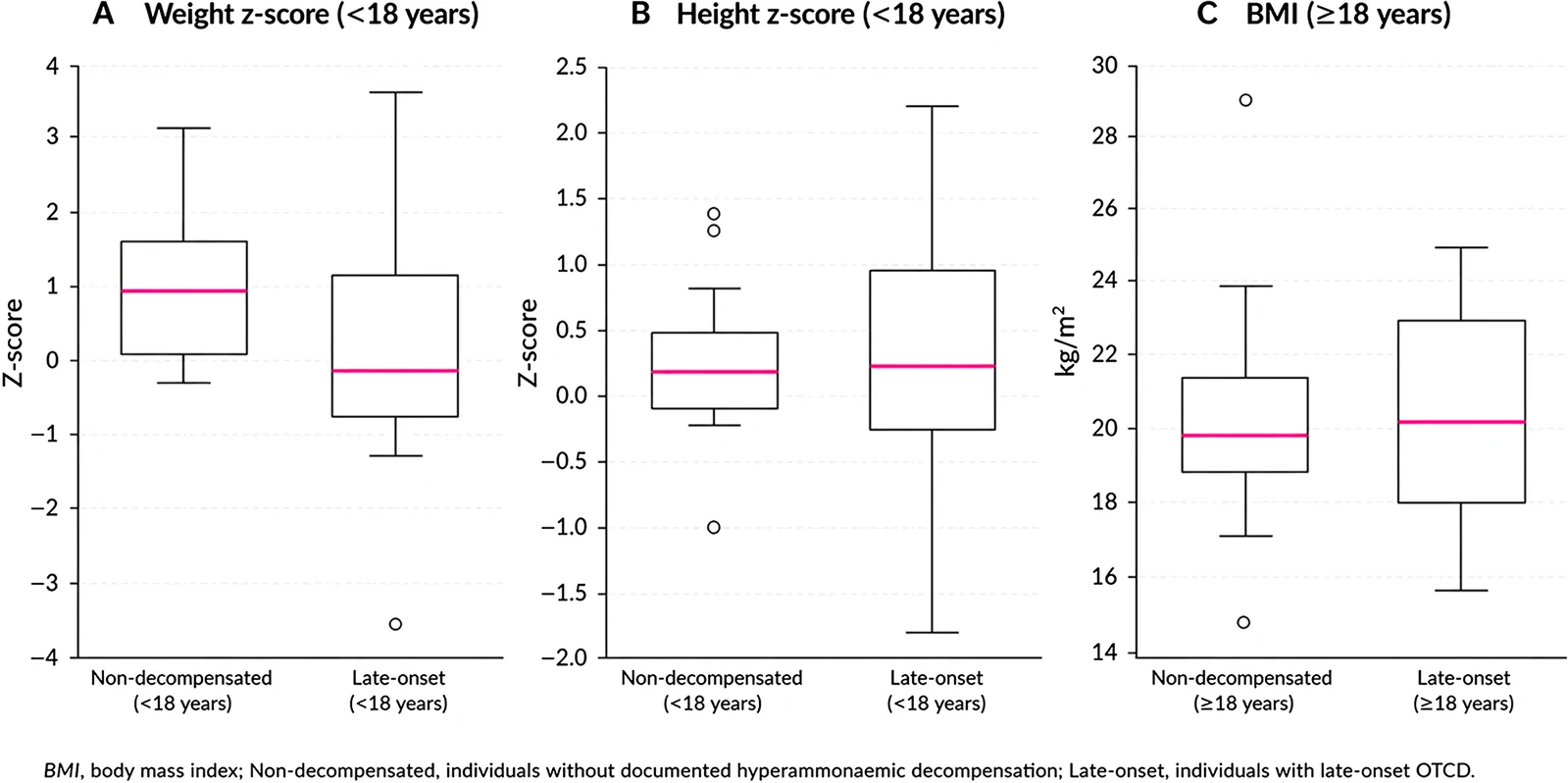
Growth parameters by phenotype and age group (Panels (left→right): paediatric weight-for-age z-score, paediatric height-for-age z-score, adult BMI). Box‑and‑whisker plots show median (centre line), interquartile range (box), and 1.5×IQR whiskers; individual points are suppressed. Paediatric metrics are weight‑ and height‑for‑age z‑scores; adult metric is BMI. Neonatal case excluded from plots. Phenotype groups: Non-decompensated and late‑onset

Phenotype-stratified anthropometrics at last follow-up are illustrated in Fig. 1. Among children, non-decompensated individuals (*n* = 9) had a mean weight-for-age z-score of 1.08 ± 1.15 and height-for-age z-score of 0.22 ± 0.64, whereas late-onset individuals (*n* = 17) had a mean weight-for-age z-score of 0.31 ± 1.75 and height-for-age z-score of 0.23 ± 1.12. Non-parametric comparisons (Mann–Whitney U test) showed no significant differences between phenotypes for weight-for-age or height-for-age z-scores (weight-for-age: *p* = 0.11; height-for-age: *p* = 0.94).

Among adults, BMI at last follow-up ranged from 16.4 to 27.7 kg/m² (median 21.55; mean 21.60 ± 3.49). Non-decompensated adults (*n* = 9) had a mean BMI of 21.41 ± 3.59, and late-onset adults (*n* = 7) had a mean BMI of 21.84 ± 3.63. BMI categories were distributed as underweight 3, normal weight 9, overweight 4, and obese 0 (non-decompensated: 2/5/2/0; late-onset: 1/4/2/0), with no significant phenotype-related differences (*p* = 0.75, Mann–Whitney U test). Adult BMI data are also presented in Fig. 1 alongside paediatric z-scores.

The neonatal patient, described separately, had a weight-for-age z-score of + 1.17 and a height-for-age z-score of − 0.72 at last follow-up and was excluded from comparative analyses.

### Neurocognitive, behavioural, and educational outcomes

Neurocognitive, behavioural, and educational outcomes were assessed in the OTCD cohort (Table 5). Formal neuropsychological assessment was undertaken in 6/26 (23.1%) individuals with late-onset disease. Among those evaluated, overall intellectual functioning ranged from average to low-average, with recurrent weaknesses observed in verbal abilities, processing speed, sustained attention, fine motor coordination, and mathematical reasoning, alongside heterogeneity across other cognitive domains.

Behavioural problems were documented in 15/26 (57.7%) individuals with late-onset disease, including attention-deficit/hyperactivity disorder (ADHD), autism spectrum disorder (ASD), combined ASD/ADHD, and selective mutism. Behavioural problems were rarely isolated, co-occurring with learning difficulties in 16/26 (61.5%) and with a history of speech delay in 10/26 (38.5%).

Behavioural abnormalities were reported in 7/18 (38.9%) non-decompensated individuals. Learning difficulties were present in 3/18 (16.7%) and speech delay in 1/18 (5.6%). These findings indicate that absence of overt hyperammonaemic admissions does not equate to absence of clinical burden.

Additional neurological comorbidities were reported in both cohorts. These were recorded in 4/26 (15.4%) individuals with late-onset disease, including seizures in 3/26 (11.5%) and migraine in 1/26 (3.8%). Among non-decompensated individuals, 4/18 (22.2%) reported recurrent headaches or migraine.

Educational trajectories are assessed by phenotype. Twenty-three of 26 (88.5%) individuals with late-onset disease were engaged in mainstream educational or vocational pathways, including school, A-levels, college, university, or employment. Among these 23 individuals, 10/23 (43.5%) required additional educational support in mainstream settings. Three of 26 (11.5%) individuals with late-onset disease attended specialist special educational needs (SEN) provision; all had multiple co-existing behavioural abnormalities and learning difficulties.

All non-decompensated individuals (18/18, 100%) followed mainstream educational or vocational pathways. Among non-decompensated adults (9/18), 4/9 (44.4%) were engaged in university-level study and 5/9 (55.6%) were in full-time employment. Notably, even non-decompensated individuals with behavioural or neurodevelopmental comorbidities demonstrated preserved functional outcomes, with successful participation in mainstream education or sustained employment.

Peak plasma ammonia concentrations at first decompensation showed wide inter-individual variability and did not demonstrate a consistent association with long-term neurocognitive outcomes. Neurodevelopmental impairment was observed across a broad range of peak ammonia values, including in individuals with only moderate hyperammonaemia, indicating that a single peak ammonia measurement does not reliably predict outcome.

In contrast, within the late-onset OTCD cohort (*n* = 26), neurocognitive morbidity was more frequent among individuals with recurrent hyperammonaemia. Sixteen individuals experienced recurrent hyperammonaemic episodes (≥ 2 episodes), of whom 15 (93.8%) exhibited neurocognitive involvement. Behavioural problems, learning difficulties, and speech delay were all substantially more prevalent among individuals with recurrent hyperammonaemia, and specialist educational provision occurred exclusively in this group.

**Table 5:** Neurocognitive, behavioural, and educational outcomes by phenotype

Outcome	Late-onset (*n* = 26)	Non-decompensated (*n* = 18)
Behavioural problems	15 (57.7%)	7 (38.9%)
Learning difficulties	16 (61.5%)	3 (16.7%)
Speech delay	10 (38.5%)	1 (5.6%)
Additional neurological comorbidities	4 (15.4%)	4 (22.2%)
Mainstream education/vocation	23 (88.5%)	18 (100%)
Additional educational support in mainstream education	10/23 (43.5%)	0/18 (0%)
SEN placement	3 (11.5%)	0 (0%)

### Neuroimaging

Brain MRI was performed in 7/26 (26.9%) individuals with late-onset OTCD (Table 6). All imaging studies were undertaken following hyperammonaemic decompensation, as part of post-encephalopathic evaluation. The dataset includes a single MRI timepoint per individual; no serial neuroimaging was available to assess temporal evolution of radiological findings.

Abnormal MRI findings were identified in 5/7 (71.4%) individuals. The abnormalities comprised chronic structural sequelae of hyperammonaemic brain injury, including gliosis, focal encephalomalacia, ulegyria, and corpus callosum involvement. The available data do not permit reliable patient-level attribution of specific lesion subtypes or detailed regional characterisation.

In this small subgroup, abnormal MRI findings co-occurred with adverse neurodevelopmental outcomes. All individuals with abnormal MRI findings (5/5, 100%) had behavioural problems and learning difficulties, and 4/5 (80.0%) had a history of speech delay. In contrast, outcomes among individuals with normal MRI findings (2/7) were heterogeneous: one individual demonstrated preserved neurodevelopment, whereas the other exhibited behavioural problems and learning difficulties despite normal imaging.

**Table 6:** Brain MRI findings of late-onset OTCD patients

Patient no.	Age at initial presentation	Age at MRI	MRI findings	Behavioural problems	Learning difficulties	Speech delay	Educational placement
16	1y 8 m	2y 10 m	Gliosis / encephalomalacia	Yes	Yes	Yes	SEN
17	3y 3 m	15y 4 m	White matter changes with callosal involvement	Yes	Yes	Yes	Mainstream
19	1y 5 m	19y 2 m	Cortical injury with ulegyria	Yes	Yes	Yes	University
41	4y	4y 4 m	Multifocal gliosis	Yes	Yes	Yes	SEN
42	8 m	11y 0 m	Encephalomalacia with callosal changes	Yes	Yes	Yes	SEN
23	4y 5 m	13y 8 m	Normal	No	No	No	Mainstream
43	1y 1 m	11y 3 m	Normal	Yes	Yes	No	Mainstream

## Discussion

This multicentre UK cohort of 45 non-transplanted individuals demonstrates that OTCD imposes substantial and sustained disease burden across the phenotypic spectrum, and that this burden is not confined to neonatal-onset disease. While neonatal OTCD remains the archetype of early severe presentation, accumulating evidence shows that late-onset OTCD can be metabolically unstable, clinically severe, and resource intensive, with recurrent hyperammonaemia, repeated hospitalisation, and enduring neurodevelopmental sequelae [9, 19, 20]. 

Diagnostic routes in our cohort reflected current practice, with approximately half identified through cascade testing and the remainder following acute hyperammonaemic presentation. However, diagnostic context alone did not appear to predict long-term trajectory in this cohort. Several individuals identified through family screening subsequently developed recurrent hyperammonaemic decompensations requiring acute and chronic intervention comparable to those diagnosed following an initial hyperammonaemic decompensation. These findings reinforce accumulating evidence that presymptomatic identification in OTCD does not ensure long-term metabolic stability, particularly in females, in whom variable hepatic X-chromosome inactivation, physiological stressors, and evolving metabolic demands influence clinical expression [21–24]. Presymptomatic diagnosis should therefore be regarded as identification of individuals at risk of future metabolic instability, necessitating structured specialist follow-up and individualised emergency planning.

Newborn screening for urea cycle disorders remains challenging because currently available biochemical markers lack sufficient sensitivity and specificity for reliable population-based screening. Emerging genomic newborn screening initiatives may provide new opportunities for earlier identification of OTCD. In our cohort, one individual was identified through the UK Newborn Genomes Programme (Generation Study), illustrating the potential of genomic approaches to detect disease before clinical presentation. Further studies are required to establish the clinical utility, cost-effectiveness, and long-term outcomes associated with genomic newborn screening strategies in OTCD and other urea cycle disorders.

Late-onset disease carried substantial cumulative metabolic burden. More than 60% of late-onset individuals in this cohort experienced hyperammonaemic decompensations after diagnosis, often recurrent and requiring repeated hospital admissions. Although the neonatal-onset case exhibited the greatest early clinical severity, individuals with late-onset disease experienced substantial longitudinal morbidity over time. Published adult and adolescent series confirm that late-onset OTCD can present with life-threatening encephalopathy, intensive care admission, and fatal outcomes when triggered by catabolic stress or intercurrent illness [18, 22, 25]. These findings indicate that later presentation does not necessarily predict a low-risk clinical trajectory.

The burden of OTCD extends beyond acute hyperammonaemic decompensations and is further amplified by the complexity of chronic management. All individuals in the decompensated cohort required long-term dietary protein restriction, yet nearly one-third of evaluable patients failed to meet age-appropriate safe protein targets despite specialist input. This reflects the recognised tension between nitrogen restriction and maintenance of adequate nutrition and growth in urea cycle disorders [12, 26, 27]. The requirement for gastrostomy feeding and totally implantable venous access devices in a substantial subset provides objective evidence of treatment intensity and healthcare utilisation in late-onset OTCD. Natural history and quality of life studies from UCDC and European cohorts further demonstrate impaired health-related quality of life across UCD severities, including individuals not classified as most severe [9, 28, 29]. 

Biochemically, our longitudinal findings suggest that Glx may represent a candidate marker of cumulative metabolic burden. Given the central role of ammonia detoxification through the glutamate–glutamine cycle, combined glutamine and glutamate (Glx) provides a biologically plausible marker of cumulative nitrogen handling that may not be captured by isolated ammonia measurements [30, 31] Higher Glx concentrations in late-onset individuals, particularly those with recurrent decompensations, are consistent with sustained glutamine accumulation as a surrogate of ongoing metabolic stress and cerebral nitrogen exposure, even when plasma ammonia is intermittently normal [2, 30, 32, 33]. However, this observational study was not designed to validate Glx as a longitudinal biomarker, and prospective protocolised studies are required to determine its clinical utility.

Neurodevelopmental morbidity was prominent in late-onset OTCD, including behavioural diagnoses, learning difficulties, and speech delay. Recurrent metabolic instability rather than peak ammonia at presentation was associated with neurodevelopmental involvement. This observation accords with previous data suggesting that cumulative hyperammonaemic burden is more relevant to neurocognitive outcome than single peak ammonia measurements [33, 34]. These findings reinforce the importance of preventing recurrent decompensation to mitigate long-term neurological impact.

Importantly, measurable morbidity was also evident in individuals classified as non-decompensated. Recurrent vomiting, chronic fatigue, behavioural symptoms, headaches, protein aversion, and dietary self-modification were common despite absence of documented hyperammonaemic admissions. These findings indicate that absence of documented hyperammonaemic decompensations does not equate to absence of clinical burden. However, the mechanisms underlying symptoms observed in non-decompensated individuals remain uncertain. Symptoms were not systematically correlated with contemporaneous biochemical abnormalities, and alternative explanations cannot be excluded. Potential contributors may include intermittent metabolic disturbances or altered nitrogen handling, but these hypotheses require prospective investigation.Recent multicentre analyses specifically evaluating females previously labelled asymptomatic demonstrate measurable neuropsychiatric symptoms and functional impact even in the absence of severe hyperammonaemic phenotypes [10]. These findings support an emerging view that OTCD manifestations may exist along a continuum rather than within a binary symptomatic/asymptomatic framework.

Beyond biochemical and neurological outcomes, OTCD imposes substantial patient and family burden. Recurrent hospitalisations, emergency treatment plans, dietary protein restriction, specialised nutritional requirements, chronic fatigue, and uncertainty regarding future metabolic stability may adversely affect education, employment, social participation, and psychological well-being. These patient-centred outcomes should be incorporated into future natural history studies and interventional trials.

The variant spectrum in our cohort is consistent with international OTCD series, in which missense substitutions predominate across phenotypic categories, whereas truncating and splice-disrupting variants are more often associated with clinically manifest disease in hemizygous males [35, 36]. The presence of the same missense variant (c.674 C > T; p.Pro225Leu) in a neonatal male and a late-onset female in our cohort further underscores the central role of X-linked inheritance and hepatic X-inactivation in modulating clinical severity, a phenomenon extensively documented in heterozygous females with OTCD [32]. These observations support a non-deterministic genotype–phenotype relationship in which clinical severity reflects the interaction between allelic effect and sex-specific modifiers [24, 37–39]. 

The recurrent variants in our cohort, particularly c.118 C > T (p.Arg40Cys) and c.240G > T (p.Lys80Asn), have both been reported in multiple independent families and are typically associated with residual enzyme activity and late-onset or milder phenotypes [40, 41]. Codon 40 substitutions, including p.Arg40Cys and p.Arg40His, affect a conserved catalytic region and have been functionally characterised as hypomorphic rather than null alleles, consistent with survival beyond the neonatal period and variable expression in heterozygous females [32, 36]. Similarly, variants affecting Arg277 (c.830G > A; p.Arg277Gln) have been repeatedly described in late-onset OTCD and in female carriers with attenuated or absent symptoms, reinforcing the concept that partial residual activity underlies delayed presentation [36, 42]. 

The identification of a regulatory/promoter variant (c.-106 C > A) in clinically stable male siblings highlights the growing recognition that non-coding *OTC* variants may reduce transcription without abolishing expression, resulting in attenuated or context-dependent phenotypes; promoter and deep intronic variants have increasingly been reported in individuals with atypical or milder biochemical profiles [43, 44]. The presence of clinically diagnosed individuals without an identifiable coding variant highlights the need for expanded molecular genetic approaches, including analysis of regulatory regions, copy-number variation, and RNA-based studies, alongside biochemical markers of urea cycle dysfunction [13, 43–45]. 

Strengths of this study include its multicentre design, longitudinal follow-up with prospective elements, and integrated analysis across clinical, biochemical, neurodevelopmental, and genetic domains. Limitations include limited representation of neonatal-onset disease due to transplantation practices, inter-centre variability in management, incomplete formal neuropsychological assessment, non-standardised biochemical sampling, and selective neuroimaging, which may introduce ascertainment bias. Furthermore, symptoms observed in non-decompensated individuals cannot necessarily be attributed exclusively to metabolic dysfunction because contemporaneous biochemical assessments were not consistently available and alternative causes were not systematically excluded. Although recurrent decompensations occurred in both sexes, our cohort was not powered for robust sex-stratified outcome comparisons.

## Conclusion

This longitudinal UK cohort demonstrates that OTCD is associated with substantial morbidity across the phenotypic spectrum. Late-onset disease was characterised by recurrent hyperammonaemic decompensations, frequent hospitalisation, and neurodevelopmental burden, while individuals without documented hyperammonaemic episodes also experienced clinically relevant symptoms and functional challenges. These findings support further investigation into mechanisms underlying ongoing clinical burden and highlight the need for improved risk stratification and development of disease-modifying therapies.

## Data Availability

The datasets generated and analysed during the current study are not publicly available because they contain potentially identifiable information from a rare disease cohort but are available from the corresponding author upon reasonable request and subject to institutional governance and data-sharing approvals.
